# Determinants of male partner involvement in antenatal care services at Kangundo Sub-County Hospital in Kenya

**DOI:** 10.4314/ahs.v22i3.11

**Published:** 2022-09

**Authors:** Pauline K Muia, Grace W Mbuthia, Rosemary K Mugambi

**Affiliations:** 1 Master of science in nursing student, Jomo Kenyatta University of Agriculture and Technology; 2 Community health department, Jomo Kenyatta University of Agriculture and Technology, P.O.BOX 6200-00200, Nairobi, Kenya; 3 Reproductive Health and midwifery department, Jomo Kenyatta University of Agriculture and Technology, P.O Box 6200-00200, Nairobi, Kenya

**Keywords:** Antenatal care services, male partner involvement, male partner accompaniment

## Abstract

**Background:**

Male partner involvement in antenatal care services is aimed at improving maternal health outcomes since men are important persons who play great roles at the family level.

**Objective:**

To assess the level and determinants of male partner involvement in antenatal care at Kangundo Sub-County hospital in Kenya.

**Methods:**

The study used analytical cross-sectional study method and was carried out in the maternal and child health clinic of Kangundo Sub-County hospital. Two hundred pregnant women at any gestational age, accompanied or not, seeking antenatal care services during the study period formed the study population. Simple random sampling technique was used to achieve a sample size of 132 participants. The data was then analyzed using the statistical package for social science (SPSS) version 20.0.

**Results:**

The study revealed a low-level male partner involvement of 34.1%. Business as male partners' occupation (OR = 2, 95% CI (0.314 – 12.729), and distance from the health facility; living 4km from the facility (OR = 5.225, 95%CI (1.319 – 20.705) and more than 5km from the facility (OR = 3.520, 95% CI (0.941 – 13.174) were significantly associated with male partner involvement.

**Conclusion:**

The factors contributing to low male partner involvement included: men being busy at work and the distance covered to reach the health facility.

## Introduction

Male partner involvement in antenatal care involves the participation of male partners in antenatal care services with the aim of improving maternal health outcomes. Male partner involved in antenatal care is governed by several factors affecting the men in the society such as social, cultural and economic factors^1^.

The need for male partner involvement in reproductive health policies was introduced in 1994 at the International Conference on Population and Development (ICPD) held in Cairo. The conference highlighted that, men are important persons who play great roles, such as being breadwinners and decision-makers in the family, thus influencing greatly on women's access to maternal health services^2^. Globally, male partners have a great role in decision-making on health at the family level^3^.

Studies have been conducted globally, with the findings revealing low male partner involvement in antenatal care. For instance, a study conducted in Southern Africa showed a low prevalence of 14%, while another study conducted in Ghana revealed a low male partner involvement of 35%^4^,^5^. In East Africa, a Ugandan-based study showed a much lower involvement of 6%, and a national survey conducted in Kenya reported a 35% male partner attendance to antenatal care^6^,^7^.

However, there are no documented studies on male partner involvement in antenatal care in Machakos county, and therefore, the study aims at assessing the level of male partner involvement, the male-related factors, and health institution factors associated with male partner involvement in antenatal care at Kangundo sub-county hospital.

## Methods

### Study design and setting

The study was carried out in the maternal and child health clinic of Kangundo sub-county hospital, whereby an analytical cross-sectional study was used to collect data.

Inclusion criteria and Exclusion criteria

Pregnant women attending the antenatal clinic at any gestational age, accompanied or not accompanied by their male partners, were included in the study. However, those less than 18 years were excluded from the study.

Study population, Sampling Technique and sample size According to the antenatal register (MOH 405), an average of 200 pregnant women were seen at the clinic monthly in the year 2019. The figure was used to estimate the target population for the study and fishers' formula with a 5% margin of error was used to determine a sample size of 132.



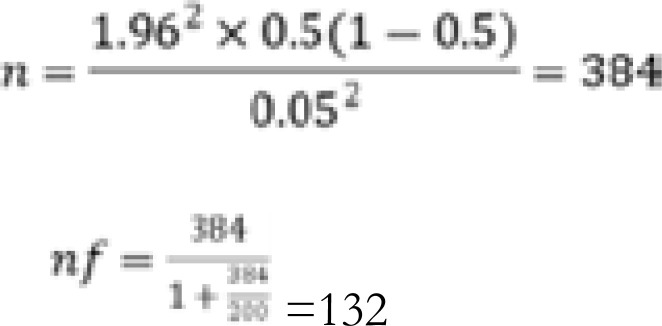



A simple random sampling technique was applied in selecting the participants at the antenatal clinic. As pregnant women streamed into the clinic each day, the procedure was explained to them then consent obtained. A box was used to mix 132 small papers marked with yes and 68 small papers marked with a no, all pregnant women meeting the inclusion criteria were randomly allowed to pick one paper. The process continued each day for a period of one month until a sample size of 132 respondents was achieved.

### Data collection

A Self-administered questionnaire with both open and closed-ended questions was used to collect data from the pregnant women over a period of one month. The questionnaire captured the socio-demographic factors of the respondents, the socio-demographic factors of the male partner, level of male partner involvement and health institution related factors. The level of male partner involvement was assessed by the use of a single open-ended question.

### Pre-test of the questionnaire

The study was pretested at the Kangundo sub-county hospital to check for ambiguities in the questions. The questionnaire was then revised to ensure that the questions were clear and understood. Pregnant women who participated in the pre-testing of the tool was not included in the actual study.

### Data Analysis and Presentation

The data collected from the questionnaire was entered and analyzed using a statistical package for social science (SPSS) version 20.0. Data analysis included both descriptive and inferential analysis. Descriptive was performed using mean (SD) and frequencies (n) as well as percentages (%). A binary Logistic regression test was performed to compare the statistical association between male partner involvement and the independent variables included in the study. Significant factors were then adjusted to control for confounding effect. Statistical significance was set at P<0.05. Results were then presented in the form of tables.

### Ethical Considerations

Permission to carry out the research was given by the Jomo Kenyatta University of Agriculture School of Nursing. Then the proposal was presented to the University of Eastern Africa Baraton, research and ethical committee for ethical clearance for conducting the study (Ref: IERC/16/06/2019). Permission was granted from National Commission for Science, Technology and Innovation (NACOSTI) and authority sought from the Kangundo sub-county hospital medical- superintendent. After which consent was obtained from the maternal and child health clinic in charge, then the respondents gave consent by signing an informed consent form.

## Results

### Socio-demographic characteristics of the respondents

The respondents' age ranged from 18–47 years. Most of the respondents (45.5%, n=60) were aged between 28–37 years, while a third of the respondents (36.4%, n=48) had attained a college level of education. Other characteristics of the respondents are shown in Table 1.

**Table 1:** Socio-demographic characteristics of the respondents

Variable	(N=132) Frequency (n)	Percentage (100%)
**Age group**		
18–27	56	42.4
28–37	60	45.5
38–47	16	12.1
**Level of education**		
Primary	23	17.4
Secondary	47	35.6
College	48	36.4
University	14	10.6
**Occupation**		
Formal employment	31	23.4
Informal employment	3	2.3
Business lady	48	36.4
Farmer	3	2.3
Not employment	47	35.6

### Level of Male Partner Involvement in Antenatal Care

**Figure 1:**
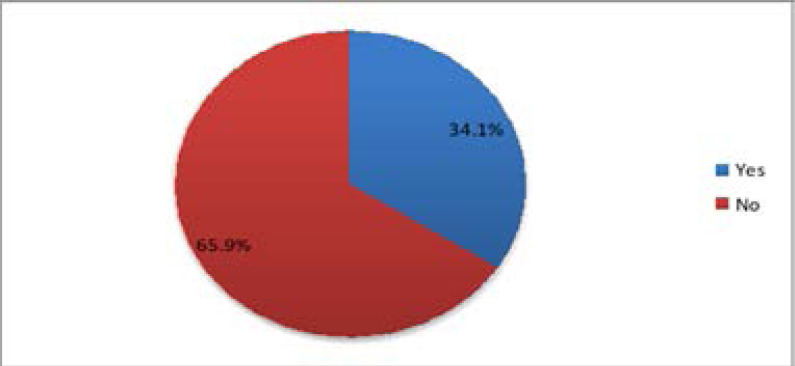
Level of partner accompaniment in the antenatal clinic

Only 34.1% (95% CI 26.1 to 42.8) of the respondents were accompanied by their male partners, while the majority 65.9% (95% CI 57.2 to 73.9) were not accompanied by their partners to the antenatal clinic in the current pregnancy as illustrated in figure 1.

Further, on reasons for non-accompaniment, a majority (62.1%, n=54) reported that their male partners were busy at work during clinic appointment days, while 10.3% (n=9) perceived pregnancy as a role for women. Other reasons for not being accompanied were not living together, lack of interest, financial constraints and fear of being seen at the clinic (table 2)

**Table 2:** Reasons for non-accompaniment to antenatal care services

Reason for not being accompanied	Frequency (n)	Percentage (%)
Busy at work	54	62.1
Financial Constraints	4	4.6
Not living together	8	9.2
Never shown interest	5	5.7
Perceived as a role for women	9	10.3
Fear of being seen at the clinic	7	8.0
Total	87	100.0

### Factors Associated with Male partner involvement in antenatal care

#### a) Male related factors and health institution factors associated with male partner involvement in antenatal care

Business as male partner occupation (p=0.049) significantly influenced male partner involvement. Businessmen were 2 times more likely to accompany their partners to antenatal care services, (OR = 2) than those who were unemployed.

Living 4kms (p=0.019) and 5kms (p=0.006) from the facility had a significant association with male partner accompaniment. Those who lived a distance of 4kms from the facility were 5 times more likely to be accompanied by their partners (OR = 5.225), while those who lived more than 5km from the facility were 4 times likely to be accompanied by their partners (OR = 3.520) as presented in Table 3.

**Table 3:** Male related factors and health institution factors associated with male partner involvement in antenatal care

Variables	Accompanied by partner N=45	Unaccompanied by partner N=87	OR	95% C.I.	p-value
**Age of partner**					
18–27 years	5 (11.1%)	18 (20.7%)	Ref		
28–37 years	22 (48.9%)	48 (55.2%)	2.4	0.481–11.97	0.29
38–47 years	14 (31.1%)	15 (17.2%)	1.455	0.373–5.679	0.59
48 years and above	4 (8.9%)	6 (6.9%)	0.714	0.166–3.075	0.65
**Partner's education**					
Primary	1 (2.2%)	4 (4.6%)	Ref		
Secondary	13 (28.9%)	39 (44.8%)	2.222	0.218–22.695	0.50
College	21 (46.7%)	26 (29.9%)	1.667	0.616–4.511	0.32
University	10 (22.2%)	18 (20.7%)	0.688	0.262–1.803	0.45
**Partner's occupation**					
Not employed	3 (6.7%)	2(2.3%)	Ref		
Formal employment	12 (26.7%)	35 (40.2%)	4.375	0.651–29.413	0.13
Informal employment	0	10 (11.5%)	0.123	0.000	0.999
Business	30 (66.7%)	40 (46.0%)	2	0.314–12.729	**0.049**
**Distance to the facility**					
Less than 1 km	9 (20%)	18 (20.7%)	Ref		
2km	16 (35.6%)	24 (27.6%)	2.2	0.681–7.103	0.19
3km	4 (8.9%)	19 (21.8%)	1.65	0.569–4.785	0.36
4km	5 (11.1%)	16 (18.4%)	5.225	1.319–20.705	**0.019**
More than 5km	11 (24.4%)	10 (11.5%)	3.52	0.941–13.174	**0.006**
**Waiting time**					
Less than	20 mins	8 (17.8%)	20 (23.0%)	Ref	
30 mins	22 (48.9%)	36 (41.4%	1.75	0.493–6.213	0.39
40 mins	8 (17.8%)	21 (24.1)	1.145	0.381–3.448	0.81
More than 60 mins	7 (15.6%)	10 (11.5%)	1.837	0.519–6.5	0.35
**Health education on male partner involvement**					
Yes	37 (82.2%)	61 (70.1%)	0.507	0.208–1.237	0.14
No	8 (17.8%)	26	29.9%)	Ref	

#### b) Adjusted Multivariate Logistic Regression Model for Factors Associated With Male Partner Involvement

After adjusting for confounding factors, the findings revealed that business as male partners occupation (AOR=4.89; P=0.031) was a significant factor associated with male partner involvement in antenatal care, as shown in table 4.

**Table 4:** Adjusted Multivariate logistic regression model for factors associated with male partner involvement

Variables	df	AOR	95% C.I.	P-value
**Partner Occupation**				
Not employed	3	Ref		
Formal employment	1	1.134	0.675–3.786	0.66
Informal employment	1	1.218	0.891–2.461	0.63
Business	1	4.89	3.78–8.541	**0.031**
**Distance to the facility**				
Less than 1 km	4	Ref		
2 km	1	1	0.561–1.541	0.24
3 Km	1	0.671	0.034–0.967	0.08
4km	1	0.984	1.346–2.651	0.13
More than 5 Km	1	1.015	0.811–3.24	0.36

## Discussion

This study found out that only 34.1% of the respondents reported having been accompanied by their male partners to the antenatal clinic. The findings concurred with an earlier study done in Kenya which showed a 35.0 % male involvement7. Similarly, earlier studies reported low prevalence of 35% and 41.4% respectively^5^,^8^. Contrary to this, a study done in Uganda found a much lower prevalence of 6 %, which was attributed to the major occupations in the study area such as trading, fishing and civil service done mostly by men6. Other studies reported higher prevalence levels of 56.9% and 70% respectively^9^,^10^.

The present study revealed that being busy at work (62.1%), perceiving antenatal care as a role for women (10.3%) and not living together (9.2%) were the top three reasons the male partners did not accompany their pregnant wives to the antenatal clinic. The findings were similar to those by a study done in Ethiopia who reported that 37.1% of male partners were working in a different town,13.6% viewed pregnancy as solely an affair for women, and 17.1% reported that pregnancy and antenatal care were not customs for men8. Additionally, a Tanzanian based study reported that 10.7% of pregnant women did not live together with their male partners while a study done in Uganda reported that 27.4% of male partners were busy at work, 19.7% viewed pregnancy and antenatal care as women's responsibility, and 14.7% reported long waiting time at the antenatal clinic as being the major reasons for non-accompaniment^9^,^6^. The similarity in all these studies could be associated with the study setting since they were all carried out in Africa.

The current study showed a significant association between male partners who were businessmen and partner accompaniment (p= 0.049). Similarly, a Kenyan based study showed a significant association between male partner employment and partner accompaniment (p= 0.001). From the odds ratio analysis, in the present study, the businessmen were 2 times more likely involved in the antenatal care services (OR 2.000) than the employed men in the previous study (OR 0.428)11. Contrary to this, a study done in Uganda did not show a significant association between male partners' employment and partner accompaniment (p= 0.622) 6. The difference in the studies could be due to the nature of men's occupations in the different study settings.

A previous study conducted in Ghana showed a significant association between the distance covered to the facility and male partner involvement in antenatal care (p=0.05) 10. Similar to the present study, which revealed that those who live 4km (p= 0.019) and more than 5km (p=0.006) from the health facility had a significant association with male partner involvement in antenatal care. Further, from the odds ratio analysis, those who live 4kms from the facility were 5 times more likely to be accompanied (OR = 5.225) while those who live more than 5kms from the facility were 4 times more likely to be accompanied by their partners (OR = 3.520). The earlier study showed that male partners were 2 times more likely to accompany their wives for antenatal care services despite the distance difficulties (OR 2.13) 10.

An earlier study conducted in Ghana revealed that waiting time at the facility was not significantly associated with male partner involvement in antenatal care (p=0.25)^5^. Similar to the present study, which did not show a significant association between waiting time at the facility and male partner involvement in antenatal care (P>0.05). Contrary, an earlier study conducted in Ghana revealed a significant association between waiting time spent at the facility and male partner involvement (p<0.001)^12^. The possiblereasons for the difference in the studies could be attributed to the study population. The earlier study was conducted on men who gave their opinion regarding the time spent at the facility, which affected their involvement in antenatal care.

The current study revealed no significant association between receiving health education and male partner involvement (p =0.14) in antenatal care. The results differed from an earlier study done in Tanzania which showed a significant association between receiving health education and male partner accompaniment 13. The possible reason for the difference could be associated with the study area. In Kenya, health education is majorly done in the hospital setting where only a few men attend, while in Tanzania, health education was done both in the hospital and in the community setting.

## Conclusion

The factors hindering male partner involvement in antenatal care services included: men being busy at work and the distance covered to reach the health facility. A future community-based study targeting the male partners is needed so as to provide more insight on the factors affecting male partner attendance in antenatal care.
